# Optimizing zebrafish rearing−Effects of fish density and environmental enrichment

**DOI:** 10.3389/fnbeh.2023.1204021

**Published:** 2023-06-29

**Authors:** Oly Sen Sarma, Natalia Frymus, Fredrik Axling, Per-Ove Thörnqvist, Erika Roman, Svante Winberg

**Affiliations:** ^1^Department of Medical Cell Biology, Uppsala University, Uppsala, Sweden; ^2^Department of Surgical Sciences, Uppsala University, Uppsala, Sweden; ^3^Department of Anatomy, Physiology, and Biochemistry, Swedish University of Agricultural Sciences, Uppsala, Sweden; ^4^Department of Pharmaceutical Biosciences, Uppsala University, Uppsala, Sweden

**Keywords:** aggression, behavior, refinement, stress, tank size, welfare

## Abstract

**Introduction:**

Despite its popularity in research, there is very little scientifically validated knowledge about the best practices on zebrafish (*Danio rerio*) husbandry, which has led to several facilities having their own husbandry protocols. This study was performed to expand knowledge on the effects of enrichment and fish density on the welfare of zebrafish, with hopes of providing a scientific basis for future recommendations and legislations.

**Methods:**

Zebrafish were reared at three different stocking densities, (1, 3 or 6 fish/L), in tanks with or without environmental enrichment. Agonistic behavior was observed twice a week for 9 weeks directly in the housing tanks. Aspects of welfare is known to be reflected in neuroendocrine stress responses. Thus, cortisol secretion in response to lowering the water level was analyzed for each group. In addition, we assessed cortisol secretion in response to confinement and risk-taking behavior (boldness) using the novel tank diving test for individual fish. At termination of the experiment fish were subjected to stress by transfer to a novel environment and brain tissue was sampled for analysis of brain monoaminergic activity.

**Results:**

Fish kept at the lowest density (1 fish/L) showed a significantly higher level of aggression than fish kept at 3 or 6 fish/L. Moreover, fish kept at this low density showed significantly higher cortisol secretion on a group level than fish kept at the higher stocking densities, when subjected to lowering of the water level. Keeping fish at 1 fish/L also had effects on brain monoamines, these fish showing higher brain dopamine concentrations but lower dopamine turnover than fish kept at higher densities. Neither stocking density or enrichment had any clear effects on the behavior of individual fish in the novel tank diving test. However, fish kept at high densities showed lower and more variable growth rates than fish kept at 1 fish/L.

**Discussion:**

Taken together these results suggest that zebrafish should not be kept at a density of 1 fish/L. The optimal stocking density is likely to be in the range of 3–6 fish/L.

## 1. Introduction

Zebrafish (*Danio rerio*) is rapidly becoming one of the most important vertebrate model organisms. Being a vertebrate, zebrafish shows strong homology with mammals including humans. Moreover, their small size (< 5 cm) allows for a reduced housing space and husbandry costs, making rearing large numbers of fish in research facilities more cost-efficient than for other vertebrates (Hill et al., 2005). To start with, zebrafish became popular as a model organism in developmental biology because of their short generation time, rapid development, and high fecundity (Hill et al., 2005; Nasiadka and Clark, 2012) in combination with large transparent embryos (Hill et al., 2005; Nasiadka and Clark, 2012). However, the use of zebrafish as a model organism is rapidly increasing also in other fields of biomedical and biological research and the use of adult fish is increasing (Choi et al., 2021), resulting in higher demand on fish rearing (Graham et al., 2018).

In nature zebrafish occur in shallow, slow-moving streams as well as in ponds that are formed alongside streams during the monsoon. These habitats are subject to dramatic seasonal changes in temperature, water levels, turbidity, predation pressure etc., all related to the monsoon (Graham et al., 2018). Zebrafish is a shoaling species, even though shoaling tendencies and shoal size varies considerably between populations. Shoaling as well as aggression and the formation of dominance hierarchies are also affected by environmental factors. The complexity of the environment appears to affect aggression and it has been reported that, in the lab, zebrafish show increased aggressive behavior in vegetated habitats (Hamilton and Dill, 2002).

At present, the laboratory housing of zebrafish is primarily focused on maintaining good hygiene and high efficiency. Fish are often reared in barren, transparent tanks with no enrichment. These conditions differ dramatically from the natural habitat of zebrafish (Graham et al., 2018; Sundin et al., 2019) and do not provide a good environment for the fish. This is likely to have negative effects on both animal welfare and the quality of the research, as a stressful environment affects the development and behavior of the fish (Graham et al., 2018). Legislation on the use of animals in scientific research and recommendations concerning rearing of zebrafish are not very detailed (2010/63/EU). Moreover, these recommendations are often arbitrary and lack scientific background (Aleström et al., 2020). This is especially apparent when it comes to recommendations on tank size and fish density (2010/63/EU). As many teleosts, zebrafish are plastic and if kept at low density, they become aggressive and form dominance hierarchies (Andersson et al., 2022). The formation of dominance hierarchies is highly stressful, especially for low-ranking subordinate fish. Thus, even though high fish densities may be stressful to the fish, low densities may be as bad since, at low densities, the level of aggression is usually high (Andersson et al., 2022). Recently, Andersson et al. (2022) reported that zebrafish kept at a 1 fish/L density had higher cortisol levels and showed elevated aggression as compared to higher densities and fish densities as high as 16 fish/L were recommended.

Schroeder et al. (2014) showed that zebrafish prefer a tank with gravel as bottom substrate if they are given a choice between gravel and a barren tank. However, when given a choice between gravel and a picture of gravel substrate, the fish were indifferent (Schroeder et al., 2014). A picture of gravel substrate is now used on a routine basis in many zebrafish facilities. Schroeder et al. (2014) also showed that zebrafish prefer an environment with artificial plants as compared to a barren tank. Environmental enrichment, in the form of plastic plants creating shelter and three-dimensional structure, has been reported to affect brain development in zebrafish (von Krogh et al., 2010; DePasquale et al., 2016). Still, this kind of environmental enrichment can also have negative effects on fish welfare. For instance, plastic plants may create hygienic problems. Another, negative effect is that they may release chemicals to the water, chemicals that may affect the fish. There are also reports showing that vegetation increases aggressive behavior in zebrafish (Bhat et al., 2015). Thus, the material and design of the enrichment is likely to be highly important. Moreover, the effects of enrichment may vary depending on fish density, since the enrichment may be monopolized by dominant fish if a strong dominance hierarchy develops (Manuel et al., 2015).

The aim of this study was to investigate effects of fish density and environmental enrichment as well as the interactions between density and enrichment, on zebrafish behavior and measures of welfare. To that end we monitored aggressive behavior and analyzed the stress-induced responses on cortisol release and brain monoaminergic activity, as well as the behavioral response to novelty of fish kept in groups at different densities with or without environmental enrichment. The results presented could be applied to formulate new and scientifically based regulations for zebrafish housing.

## 2. Material and methods

### 2.1. Fish and experimental groups

Zebrafish larvae of the AB strain were purchased from the Zebrafish core facility at Karolinska Institutet, Solna, Sweden, in August of 2019. All zebrafish were born on the same day (28 August 2019). The purchased larvae were transported to the Behavioral Neuroendocrinology lab at Uppsala University in October of 2019 and upon arrival they were placed in 9.5 L tanks in a stand-alone system (Aquaneering, San Diego, 117 USA) supplied with recirculating copper-free Uppsala municipal tap water (10% daily exchange). Temperature was maintained at 27.0 ± 1.5°C and the photoperiod was 14L:10D (lights on at 07:00 h). Animals were fed with a combination of granulated food (Sparos I&D, Olhão, Portugal) and rotifer culture twice a day. On October 28, experimental groups were generated by transferring fish to new 9.5 L tanks equipped with: (1) a picture of gravel as bottom substrate [picture used by Schroeder et al. (2014)], (2) an artificial plant rooted to the bottom of the tank, (3) the picture of bottom substrate and an artificial plant, or (4) barren tanks without any enrichment (Supplementary Figure 1). Moreover, the fish were kept in these environments at three different densities: 10 fish, 30 fish or 60 fish per tank, representing a density of 1, 3 or 6 fish/L. Three replicates per treatment were used, i.e., a total of 48 experimental groups. The placement of the groups in the stand-alone system was randomized. At the end of the experiment fish were euthanised, weighed for body mass (g) and measured for total length (cm). Sex was determined by dissection.

### 2.2. Ethical considerations

The study was authorized by the Uppsala Animal Ethical Committee (permit 5.8.18-10125/2018). Before arriving to Uppsala University, the permit was held by the Zebrafish core facility at the Karolinska Institutet, Solna, Sweden (permit N220/15). All followed the guidelines of the Swedish Legislation on Animal Experimentation (Animal Welfare Act SFS 2018:1192) and the European Union Directive on the Protection of Animals Used for Scientific Purposes (Directive 2010/63/EU).

### 2.3. Observation of agonistic behavior

Starting one week after the establishment of the experimental groups, the behavior of the fish in the experimental groups was monitored. Each tank was observed independently for 5 min, two times a week for a total of 9 weeks. Because of the large number of tanks, each weekly observation was split into 2 days with half of the groups being observed on the first day and the other half of the groups on the second day. Observations were performed between 10:00 AM and 4:00 PM by the same observer. These behavioral observations were conducted during three periods: (1) observation of juvenile fish for 5 weeks (4 November to 8 December 2019), (2) observation of adult fish for 2 weeks (6 January to 17 January 2020), and (3) continuation of observation of adults for another 2 weeks (16 March to 27 March 2020).

Each tank was observed from a distance of approx. 50 cm. Frequency of the agonistic behaviors exhibited by the fish were quantified by manual observations according to an ethogram (Supplementary Table 1; Kalueff et al., 2013).

### 2.4. Cortisol secretion in response to acute stress on a group level

Analysis of cortisol secretion on a group level was performed non-invasively by measuring the amount of cortisol released in the water at 8 months post-hatching. The fish were subjected to confinement stress by lowering the water level in the holding tank to a depth of 1 cm for 30 min. After the confinement period the tank was refilled with fresh system water and a water sample of 500 mL was taken, using a silicon hose, immediately after refilling. Additionally, on each day of sampling, 2 samples of recirculating water were collected as a reference for background cortisol levels in the recirculating water. Cortisol secretion was expressed as ng cortisol/fish and h.

### 2.5. Cortisol secretion in response to acute stress in individual fish

At 9 months post-hatching, two fish from each tank (96 fish in total) were individually exposed to a confinement stress in a 50 mL Falcon tube for 30 min. Next, the fish were released into oxygenated tanks and the water sample from the Falcon tubes was collected and later analyzed for cortisol levels. In this part 17 out of 96 samples were lost due to technical problems. There were only two experimental groups with no missing data (gravel 10 and gravel 30) whereas in one group (plant 30) half of the samples were lost, leaving 4 samples (one from each replicate) for analysis. In the rest of the groups 1–2 samples were lost per group.

After the individual confinement stress, fish were put in a recovery tank for 10 min and were then anesthetised in 10% benzocaine (6 ml in 300 ml tap water), tagged by visual implant elastomers (VIE) and returned to their holding tanks. In the novel tank diving test (see below), performed 18–27 days later, the behavior of these fish was screened to study effects of a previous stress on the behavior of fish from different rearing conditions.

### 2.6. Extraction and analysis of water cortisol

Cortisol levels from the water samples were extracted using C18 solid-phase extraction columns (Hypersep C18, Thermo Scientific, Rockwood, TN, USA). Solid-phase columns were eluted with ethyl acetate and subsequently evaporated using a stream of nitrogen. The solid rest was re-suspended in a 1 mL 1:1 mixture of MilliQ-H2O and isopropanol, vortexed, and spun down; thereafter, empty glass vials were washed with 500 μL of isopropanol (to extract any cortisol stuck to the glass), which was transferred to the samples. Samples were evaporated using a Speed-Vac until dry, re-suspended in 32 mL of MilliQ-H2O, and stored at +4°C.

The extracted cortisol samples were analyzed by liquid chromatography-mass spectrometry (LC-MS) using a Waters Accuracy UPLC-MS system (Milford, MA, USA). Cortisol standards were prepared by using 50 M Hydrocortisone solution (Sigma-Aldrich, Saint Louis, MO, USA) diluted into concentrations between 0.08 and 2 M together with a blank standard of MilliQ-water (for details, see Supplementary Information).

### 2.7. Novel tank diving test

Two weeks after the acute stress test on individual fish, a behavioral screening of individual fish from the different rearing environments was performed, using the novel tank diving test (NTDT). Two randomly selected fish from each rearing tank were tested. In addition, the fish previously used for studies on acute stress response were identified by elastomer tags and tested (4 fish in total from each tank). The novel tank consisted of a narrow and deep 1.8 L rack system tank (length top 26 cm, length bottom 23 cm, width 5 cm and depth 12 cm; Aquaneering, USA) filled with fresh Uppsala municipal tap water (26–28°C). A fish was placed in the tank and allowed to explore it for 10 min. Four individuals were tested simultaneously in four different arenas. An infrared light board (Noldus, Wageningen, Netherlands) was placed behind the tanks and infra-red camera (JVC Super LoLux, Thailand) recorded the diving arenas from the side. Two photographic lights (Walimex daylight 1000, Netherlands) provided moderate lighting. To avoid disturbance, the experimenter was not present in the room during video recordings. The video tracking software Ethovision XT14 (Noldus, Wageningen, Netherlands) was used to record the number of visits, duration (s and %) of time spent, mean velocity (cm/s) and total distance (cm) moved in the bottom, middle and top third of the tank, as well as mean velocity (cm/s) and total distance (cm) moved in the arena. Total activity was calculated as the sum of frequencies to the three zones. Tracks were manually edited to correct any tracking errors caused by reflections of visible light. Following the NTDT, the fish were returned to their rearing tanks.

### 2.8. Termination of the experiment and tissue sampling

The experiment was terminated 10 months post-hatching. Four zebrafish were randomly captured from tanks with 1 fish/L and six fish from groups with a density of 3 and 6 fish/L. Half of the fish were directly euthanized in a water bath containing an overdose of 1 g/L benzocaine and sampled. The other half of the fish were transferred to separate new unfamiliar barren 9.5 L tanks. The transfer to an unfamiliar tank served as an acute stressor and the fish were euthanized in a water bath containing an overdose of 1 g/L benzocaine and sampled after spending 30 min in the novel tank. The brains were dissected into forebrain (telencephalon and diencephalon) and brain stem and immediately placed in dry ice and stored at −80°C. The brain stems were analyzed for tissue levels of monoamines and monoamine metabolites.

### 2.9. Analysis of brain monoamines and monoamine metabolites

The frozen brain tissue samples were homogenized with 250 μL acetate buffer (pH 5) containing 10 ng/ml dihydroxybenzoic acid (DHBA) (Mustafa et al., 2019) using a Sonifier cell disruptor B-30 (Branson Ultrasonics, Danbury, CT, USA). DHBA was used as internal standard, and a standard solution with a known concentration (10 ng/ml) of all monoamines was used to compare the content in the brains. The homogenized samples were centrifuged at 20,000 *g* and 4°C for 10 min and the supernatant was used for the HPLC-EC analysis. The supernatant was used for high performance liquid chromatography with electrochemical detection (HPLC-EC), analyzing the monoamines dopamine (DA) and serotonin (5-hydroxytryptamine, 5-HT), as well as the DA metabolite 3,4-dihydroxyphenylacetic acid (DOPAC) and the 5-HT metabolite 5-hydroxyindoleacetic acid (5-HIAA), as described by Øverli et al. (1999). In short, the HPLC-EC system consisted of a solvent delivery system model 582 (ESA, Bedford, MA, USA), an autoinjector Midas type 830 (Spark Holland, Emmen, Netherlands), a reverse phase column (Reprosil-Pur C18-AQ 3 μm, 100 mm × 4 mm column, Dr. Maisch HPLC GmbH, Ammerbuch-Entringen, Germany) kept at 40°C and an ESA 5200 Coulochem II EC detector (ESA, Bedford, MA, USA) with two electrodes at reducing and oxidizing potentials of −40 mV and +320 mV. A guarding electrode with a potential of +450 mV was employed before the analytical electrodes to oxidize any contaminants. The mobile phase consisted of 75 mM sodium phosphate, 1.4 mM sodium octyl sulfate and 10 μM EDTA in deionised water containing 7% acetonitrile brought to pH 3.1 with phosphoric acid. Samples were quantified by comparison with standard solutions of known concentrations. DHBA was used as internal standard to correct for recovery using HPLC software Clarity™ (DataApex Ltd., Prague, Czech Republic). The ratios of 5-HIAA/5-HT and DOPAC/DA were calculated and used as an index of serotonergic and dopaminergic activity, respectively.

For normalization of brain monoamine levels, brain protein weights were determined with Bicinchoninic acid protein determination (Sigma Aldrich, Sweden) according to the manufacturer’s instructions. The assay was read on a Qubit^®^ 2.0 Fluorometer (Ref: Q32866, Invitrogen, Life Technologies Holdings Pte Ltd., Singapore) at a wavelength of 570 nm.

### 2.10. Statistical analyses

For agonistic behavior the fight scores from both days of observation were summed up for each tank creating a weekly aggression score per 10 min observation. Weekly aggression scores were analyzed using a two-way repeated measure ANOVA, with time (observation week) as within-subject factor and density (1, 3 or 6 fish/L) and treatment (enrichment in the form of gravel picture, plant, a combination of both or barren) as between-subject factors. Significant effects were further analyzed by two-way and one-way ANOVAs to reveal main effects and the Bonferroni test was used for *post hoc* comparisons.

Data on body mass, body length and Fulton K (Froese, 2006) were analyzed using a three-way ANOVA with sex, enrichment and stocking density as between-subject factors. Significant effects were further analyzed by two-way and one-way ANOVAs to reveal main effects. The Bonferroni test was used for *post hoc* comparisons. Coefficients of variance (CV) were calculated for body mass and body length, and effects of sex, enrichment and stocking density were evaluated in a similar way using ANOVA. Sex ratio (males/females) was calculated separately for each holding tank and effects of enrichment and stocking density were evaluated using a two-way ANOVA. In a similar way, data on cortisol secretion rate were analyzed by a two-way ANOVA and the Bonferroni test was used for *post hoc* comparisons.

Data on cortisol release were analyzed by two-way ANOVA with density stocking density and enrichment as between subject factors. Significant effects were further analyzed by one-way ANOVA to reveal main effects and the Bonferroni test was used for *post hoc* comparisons.

Behavioral data from the NTDT and data on brain tissue concentrations of DA, 5-HT, DOPAC and 5-HIAA, as well as DOPAC/DA and 5-HIAA/5-HT ratios, did not fulfill the assumption of normal distribution. Since we were unable to achieve normal distribution by transformation, we analyzed these data using non-parametric Kruskal-Wallis ANOVA followed by Mann-Whitney U-test.

Data were considered statistically significant at *p* < 0.05. IBM SPSS statistics (version 28.01.01) was used for statistics and graphs were created using GraphPad Prism [Version 9.3.1 (350) for Mac, GraphPad Software, San Diego, CA, USA].

## 3. Results

### 3.1. Sex ratio, body length, body mass and condition factor (Fulton K) of the fish

**FIGURE 1:**
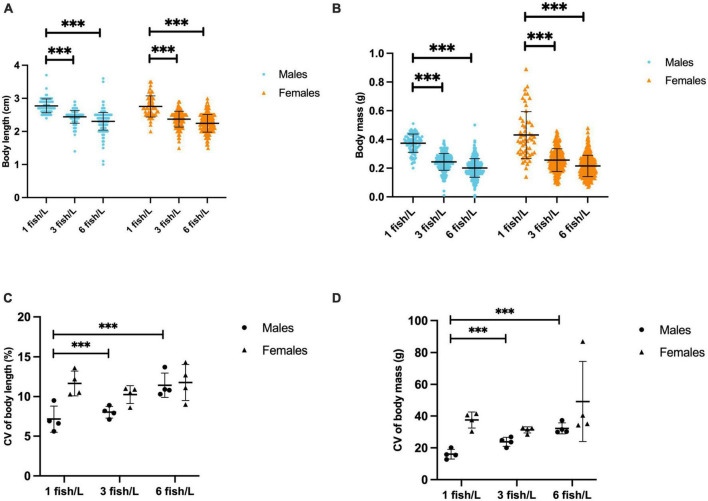
Total body length (cm, **A**), body mass (g, **B**), coefficient of variance (CV) of body length **(C)** and CV of body mass **(D)** of male and female zebrafish kept at different stocking densities, 1 fish/L (*n* = 148), 3 fish/L (*n* = 442) or 6 fish/L (*n* = 852). Values are shown as individual fish **(A,B)** and individual tanks **(C,D)** with mean marked as line and SD in whiskers. ***p < 0.001 compared to fish kept at 3 or 6 fish/L.

The sex ratio was skewed with a higher frequency of males than females (Table 1) but there were no effects of treatment, neither enrichment [F(3, 36) = 0.342; *p* = 0.795] nor density [F(2, 36) = 0.345; *p* = 0.711]. At the termination of the experiment there were significant effects of fish density on body mass [F(2, 1,243) = 241.578; *p* < 0.0001] and total length [F(2, 1,243) = 199.818; *p* < 0.0001], but not on Fulton K [F(2, 1,243) = 1.643; *p* = 0.194]. There was also a significant interaction effect of sex and density on body mass [F(2, 1,243) = 3.081; *p* < 0.05], but not on body length [F(2, 1,243) = 0.501; *p* = 0.606] or Fulton K [F(2, 1,243) = 1.214; *p* = 0.297]. Fish at the lower density of 1 fish/L were longer (Figure 1A) and had higher body mass (Figure 1B) than fish kept at higher density, i.e., 3 or 6 fish/L. Coefficients of variance (CV) were calculated to evaluate treatment effects on the variance in body length (Figure 1C) and body mass (Figure 1D). The results showed that there were significant effects of fish density on both CV for body mass [F(2, 47) = 5.167; *p* = 0.011] and CV for body length [F(2, 47) = 14.114; *p* < 0.0001], but no effect of enrichment [F(3, 47) = 0.909; *p* = 0.446 and F(3, 47) = 0.601; *p* = 0.618 for CV for body mass and CV for body length, respectively]. However, there was a significant interaction effect of density and enrichment on body length [F(6, 47) = 2.663; *p* = 0.031], fish reared with enrichment showing smaller CV for body length than fish kept in tanks without enrichment (Supplementary Table 2).

**TABLE 1:** Sex ratio of zebrafish (n_total_ = 1,442, n_male_ = 934, n_female_ = 508) kept at different stocking densities with and without environmental enrichment.

	1 fish/L				3 fish/L				6 fish/L			
**Enrichment**	**Bar**	**GP**	**GPL**	**PL**	**Bar**	**GP**	**GPL**	**PL**	**Bar**	**GP**	**GPL**	**PL**
Sex ratio (M/F)	1.29	1.79	2.45	1.46	1.76	1.73	1.41	1.30	1.92	2.03	1.99	2.32

Bar, barren tank; GP, gravel picture; GPL, gravel picture and plastic plant; PL, plastic plant.

### 3.2. Agonistic behavior

**FIGURE 2:**
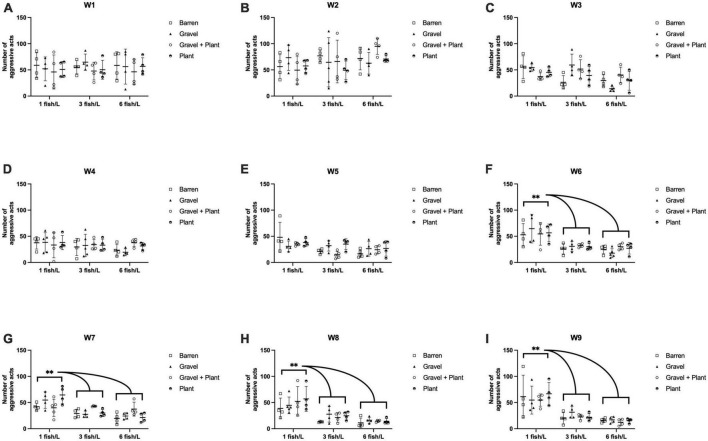
The number of aggressive acts per week performed by zebrafish during 9 weeks (W1–W9, **A–I**) of rearing at different stocking densities with or without environmental enrichment. **p < 0.01 compared to fish kept at 3 or 6 fish/L. Values are shown as individual tanks with mean marked as line and SD in whiskers.

Various aggressive actions were observed, but the majority of the noted aggressive behaviors consisted of strikes and chases. Circling was observed occasionally, especially in the first few weeks after housing. To evaluate the importance of the plant as an enrichment the number of animals attempting to hide in or behind a plant was noted. However, in the duration of the whole observation period, only nine instances of active hiding were noted. The weekly aggression scores ranged from 2 to 124. The weekly aggression scores for groups kept at different densities and with or without environmental enrichment is presented in Figure 2.

A repeated measure two-way ANOVA was performed to evaluate the effects of enrichment, density and time on aggression score. This analysis showed that there was a significant effect of time [F(7.78, 272.19) = 30.56, *p* < 0.001] and a significant interaction effect of time and density [F(15.55, 272.19) = 6.22, *p* < 0.001] but no interaction between time and enrichment [F(23.33, 272.19) = 0.72, *p* = 0.827] or time, enrichment and density [F(46.66, 272.19) = 0.98, *p* = 0.516]. There was a significant between subject effect of density [F(2, 35) = 23.41, *p* < 0.001] but not of enrichment [F(3, 35) = 0.31, *p* = 0.816] and no interaction between density and enrichment [F(6, 35) = 1.66, *p* = 0.159]. *Post hoc* analysis showed that the aggression score was significantly higher in fish kept at a stocking density of 1 fish/L than in fish kept at 3 fish/L (*p* < 0.001) or 6 fish/L (*p* < 0.001). There was no difference in aggression score between fish kept at 3 and 6 fish/L (*p* = 0.184).

### 3.3. Cortisol secretion in response to group confinement

**FIGURE 3:**
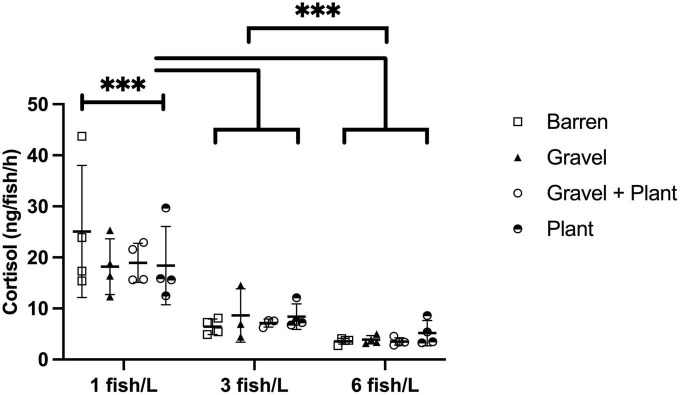
Cortisol secretion to surrounding water by zebrafish kept at different stocking densities with or without environmental enrichment. Groups of fish were subjected to confinement by lowering of the water level to 1 cm for 30 min. Values are shown as individual tanks with mean marked as line and SD in whiskers. ***p < 0.001 compared to fish kept at 3 or 6 fish/L.

The amount of cortisol extracted from each tank following group confinement was used to calculate the cortisol secretion rate per fish and hour. This rate ranged from 2.7 to 43.7 ng/fish/h. The cortisol secretion rate for groups kept at different densities and with or without environmental enrichment is presented in Figure 3.

There was a statistically significant effect of stocking density [F(2, 34) = 98.81, *p* < 0.001] but no effect of enrichment [F(3, 34) = 0.34, *p* = 0.796] and no interaction between enrichment and density [F(6, 34) = 0.79, *p* = 0.587].

*Post-hoc* tests revealed that fish kept at 1 fish/L released significantly more cortisol than fish kept at 3 (*p* < 0.001) and 6 fish/L (*p* < 0.001). There was also a difference in cortisol release between fish kept at 3 and 6 fish/L (*p* < 0.001), fish kept at 6 fish/L showing significantly lower cortisol release.

### 3.4. Cortisol secretion in response to individual confinement

**FIGURE 4:**
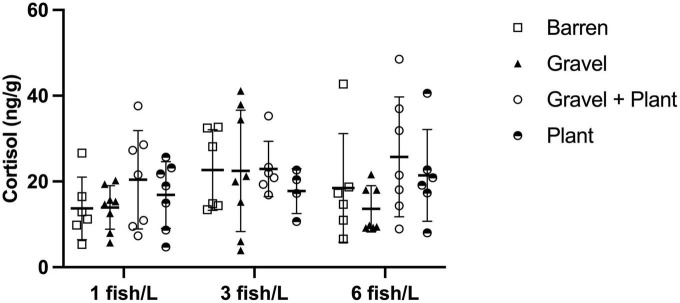
Cortisol secretion after confinement stress in 50 mL Falcon tube for 30 min by individual zebrafish kept at different stocking densities with or without environmental enrichment. Values are shown as individual fish with mean marked as line and SD in whiskers.

The amount of cortisol extracted from water samples collected after the confinement of individual fish was used to calculate the cortisol secretion rate per gram fish and hour for each individual. This rate ranged between 3.9 and 72.4 ng/g/h. The cortisol secretion rate for individual fish from different densities and with or without environmental enrichment is presented in Figure 4.

There was no significant interaction between density and enrichment on the cortisol secretion rate in individual zebrafish [F(3, 71) = 0.479, *p* = 0.698]. Neither were there any significant main effects of either density [F(1, 71) = 0.157, *p* = 0.693] or enrichment [F(1, 71) = 1.121, *p* = 0.346].

### 3.5. Novel tank diving test

The results from the NTDT are shown in Supplementary Tables 3–5.

There were significant effects of fish density on frequency in the bottom zone (*H* = 8.726, *p* = 0.016), frequency in the middle zone (*H* = 6.261, *p* = 0.044) and total activity in the arena (*H* = 6.326, *p* = 0.042) in the NTDT. Fish kept at 1 fish/L showed higher total activity and were more frequent in the bottom and middle zones than fish kept at 3 and 6 fish/L (Supplementary Table 3). Enrichment, on the other hand, had no significant effect on any of the behavioral variables of the NTDT (Supplementary Table 4).

In addition to the effects of stocking density and enrichment we also studied the effects of prior stress on the behavior of fish from these different holding conditions by screening the behavior of the two fish from each holding tank that had been subjected to confinement stress and tagged 18–27 days prior to the NTDT. This previous stress experience was found to have a clear effect on the behavior of the fish (Supplementary Table 5), stressed fish spending less time (*U* = 3,183, *p* = 0.015) and shorter percentage duration (*U* = 3,183, *p* = 0.015) at the bottom than non-stressed fish. Also, stressed fish had a longer distance moved in the middle (*U* = 3,001, *p* = 0.003) and top zone (*U* = 3,153, *p* = 0.012), as well as higher frequency in middle (*U* = 3,192, *p* = 0.016) and top (*U* = 3,027, *p* = 0.004) zones, and longer duration in these upper zones (middle zone *U* = 3,032, *p* = 0.004; top zone *U* = 3,027, *p* = 0.004). Overall, stressed fish were more active as indicated by a higher total activity (*U* = 3,207, *p* = 0.013) compared to non-stressed fish (Supplementary Table 5).

### 3.6. Monoamines

The results from the analyses of monoamines are shown in Tables 2–4.

**TABLE 2:** Levels of monoamines, metabolites and ratios between metabolite and monoamine in the hindbrain of stressed and unstressed zebrafish.

	DA	DOPAC	DOPAC/DA	5-HT	5-HIAA	5-HIAA/5-HT
Stressed (*n* = 127)	5.7 (6.7)	2.0 (1.8)**	0.281 (0.3)***	8.4 (3.9)	5.2 (3.4)***	0.624 (0.3)
Unstressed (*n* = 128)	5.0 (5.1)	1.4 (0.9)	0.214 (0.3)	7.7 (4.0)	2.3 (1.8)	0.301 (0.1)

Values represent median with interquartile range in brackets. **p < 0.01, ***p < 0.001 compared to unstressed fish (Mann–Whitney U-test).

**TABLE 3:** Levels of monoamines, metabolites and ratios between metabolite and monoamine in the hindbrain of zebrafish kept at different stocking densities.

	DA	DOPAC	DOPAC/DA	5-HT	5-HIAA	5-HIAA/5-HT
1 fish/L (*n* = 63)	7.3 (5.8)**	1.7 (1.5)	0.199 (0.2)#	8.8 (4.2)	3.6 (3.0)	0.366 (0.3)
3 fish/L (*n* = 96)	4.9 (5.5)	1.7 (1.4)	0.245 (0.3)	8.1 (3.7)	3.5 (3.8)	0.458 (0.4)
6 fish/L (*n* = 96)	4.7 (4.9)	1.6 (1.4)	0.247 (0.3)	7.6 (4.2)	3.3 (3.1)	0.392 (0.3)

Values represent median with interquartile range in brackets. **p < 0.01 compared to fish kept at 3 or 6 fish/L; #*p* < 0.05 compared to fish kept at 3 fish/L (Mann–Whitney U-test).

**TABLE 4:** Levels of monoamines, metabolites and ratios between metabolite and monoamine in hindbrain of female and male zebrafish.

Sex	DA	DOPAC	DOPAC/DA	5-HT	5-HIAA	5-HIAA/5-HT
Female (*n* = 93)	5.1 (6.0)	1.6 (1.6)	0.261 (0.4)	7.5 (3.9)	2.9 (3.1)	0.376 (0.3)
Male (*n* = 162)	5.7 (5.6)	1.7 (1.3)	0.227 (0.2)	8.6 (4.3)*	3.9 (3.6)**	0.469 (0.4)*

Values represent median with interquartile range in brackets. *p < 0.05, **p < 0.01 compared to females (Mann–Whitney U-test).

At the termination of the experiment half of the fish were stressed by transfer to a novel tank whereas the rest of the fish were sampled directly from the holding tank. Stressed fish showed significantly higher brain tissue levels of DOPAC (*U* = 4,229, *p* < 0.001) and 5-HIAA (*U* = 2,163, *p* < 0.0001) as well as elevated 5-HIAA/5-HT (*U* = 768, *p* < 0.0001) and DOPAC/DA (*U* = 4,224, *p* < 0.05) ratios as compared to fish sampled directly from the holding tank (Table 2).

There were no significant effects of enrichment on either of the monoamines, metabolites or ratios (data not shown). However, stocking density had significant effects on brain levels of DA (*K* = 15.532, *p* < 0.001) and DOPAC/DA ratios (*K* = 8.477, *p* = 0.014), fish kept at the lowest density (1 fish/L) showing higher DA concentrations than fish kept at 3 fish/L (*K* = 38.455, *p* = 0.001) or 6 fish/L (*K* = 36.405, *p* = 0.002). Moreover, fish kept at 1 fish/L showed lower DOPAC/DA ratios than fish kept at 3 fish/L (*K* = −2.817, *p* = 0.015), whereas there was no difference in DOPAC/DA ratios between fish kept at 1 fish/L and fish kept at 6 fish/L (*K* = 0.699, *p* = 1.000) or between fish kept at 3 and 6 fish/L and (*K* = −2.051, *p* = 1.000; Table 3).

Finally, we detected significant effects of sex on brain concentrations of 5-HT (*U* = 7,922, *p* = 0.020), and 5-HIAA (*U* = 8,300, *p* = 0.002) as well as on the 5-HIAA/5-HT ratio (*U* = 7,662, *p* = 0.014), with males showing higher 5-HT and 5-HIAA concentrations, and also higher 5-HIAA/5-HT ratio, than females (Table 4).

## 4. Discussion

It is often assumed that high fish density results in a welfare problem in fish rearing units and that the fish would benefit from being kept at lower density. Still, previous results are ambiguous with studies showing elevated aggression and stress at high densities (Spence et al., 2008; Gronquist and Berges, 2013), whereas others show the opposite results (Pavladis et al., 2013; Andersson et al., 2022). The result of this study clearly show that the level of aggressive behaviors was significantly higher in groups with 1 fish/L as compared to groups with 3 and 6 fish/L. There was no difference in aggressive behavior between the groups kept at the two higher densities. If the number of aggressive acts is calculated per fish the difference between the lowest and the two higher densities became even more striking. Thus, socially subordinate fish in groups with a density of 1 fish/L are likely to have received high numbers of aggressive acts, resulting in chronic social stress.

Aggression changed over time and during the first weeks. Aggression was similar at all three fish densities but after 5 weeks the level of aggression decreased in groups with 3 and 6 fish/L whereas it remained at the same high level at the lowest density (1 fish/L). This change in aggression over time could be due to the establishment of stable dominance hierarchies. However, in the present study the number of aggressive acts reported are the total number of aggressive acts in the group which makes it impossible to determine social rank of individual fish or the stability of dominance hierarchies. Tagging in zebrafish, especially tagging with external tags, is invasive (Dahlbom et al., 2011) and not possible for this kind of behavioral studies with relatively large groups of fish. However, when zebrafish are kept in smaller groups the social structure is often characterized by one fish being dominant and despotic, attacking the rest of the group members at an equal frequency (Paull et al., 2010). Thus, there will be one, or sometimes a few, highly dominant despots dominating the rest of the group members, which will all be subordinate and equally stressed. When kept in larger groups aggression is first of all diluted, since the number of aggressive dominant individuals is still limited but the number of low-ranking fish being subjects for the aggression from these aggressive tank mates is large. Zebrafish also show considerable behavioral plasticity and in larger groups they often form shoals, at least if the tank size allows, even though shoaling and aggression varies between different zebrafish populations (Graham et al., 2018).

When subjected to confinement in groups, fish kept at the lowest density (1 fish/L) showed higher cortisol release to the surrounding water than fish kept at the higher densities. Moreover, there was also a difference in cortisol release between fish kept at 3 and 6 fish/L, fish kept at 3 fish/L showing higher cortisol release. Water cortisol, as analyzed in this study, represents the integrated response, i.e., the area under the response curve, which is likely to be a better indicator of fish welfare than plasma cortisol levels at one specific time point (Koolhaas et al., 2011). In animals subjected to chronic stress and poor welfare, the time course of the hypothalamus-pituitary-adrenal/hypothalamus-pituitary-interrenal axis activation has been shown to be shifted, chronic stress resulting in a slower elevation as well as a slower recovery of plasma cortisol following stress (Koolhaas et al., 2011). Thus, the elevated cortisol response that we observed in fish kept at the lower densities supports the suggestion that zebrafish welfare at this low density is compromised by chronic social stress. However, we did not find any difference between fish kept at different densities when individual fish were subjected to confinement stress. It is difficult to speculate about the cause of this difference between the two stress paradigms applied, even though it is likely that the challenge experienced by the fish under these different conditions was very different. Confinement on a group level is likely to be a more intense stressor. When in groups, confinement was achieved by lowering the water level in the tank to only one cm. In nature zebrafish is subjected to avian predation (Spence et al., 2006). Thus, shallow water, especially without any possibility to hide or escape represents a high-risk environment. The small water volume left in the tank also resulted in crowding, and the panic behavior of the tank mates may have made the situation even more stressful. For individual confinement, individual fish were netted and transferred to Falcon tubes in which they were kept for 30 min, the same time as used for group confinement. It is difficult to compare cortisol release data from different studies since stress protocols as well as methods for analysis differ but the cortisol release rate observed for zebrafish during group confinement appear very high whereas the cortisol release by individually confined fish are more in line with previously published data (e.g., Pavladis et al., 2013). Interestingly, subjecting the fish to individual confinement appeared to have striking effects on the behavior in the NTDT, stressed fish being more active and spending more time in the upper part of the test arena than non-stressed. Normally, this would be interpreted as a more bold and less neophobic behavior (Fontana et al., 2022). Still, in this case we find it more likely that it is more related to panicking and escape attempts by the fish. Fish kept at 1 fish/L were more active in the bottom and middle zones of the NTDT arena than fish kept at higher stocking density. This difference in activity is difficult to interpret but may suggest a more a more risk aversive behavior of fish kept at a stocking density of 1 fish/L, chronic stress being known to result in neophobia and risk aversive behavior (Marcon et al., 2018).

In previous studies on the effects of fish density on zebrafish welfare, fish have been sampled directly from the holding tanks and cortisol analyzed from whole body extraction (Ramsay et al., 2006; Pavladis et al., 2013; Ribas et al., 2017). These studies show clear effects of crowding, i.e., fish held at high density showed elevated whole-body cortisol. However, in these studies zebrafish have been kept at much higher density than in our study. In agreement with our results, Pavladis et al. (2013) observed that if kept at low densities zebrafish developed strong dominance hierarchies that compromised fish welfare but at the same time, they reported that high densities resulted in crowding and elevated whole-body cortisol. The high densities used in their study was higher than densities in our study and it seems that fish densities of 3–6 fish/L may be optimal for zebrafish, even though optimal densities will probably be affected by other factors such as tank size (Pavladis et al., 2013), background color (Pavladis et al., 2013; de Abreu et al., 2020) and enrichment (Wilkes et al., 2012; Schroeder et al., 2014).

Stress is known to result in an elevation of brain serotonergic activity (Summers and Winberg, 2006; Backström and Winberg, 2017). As expected, stress, induced by transferring the fish to a novel tank, resulted in an activation of the brain 5-HT and DA systems, as indicated by elevated brain levels of 5-HIAA and DOPAC as well as elevated 5-HIAA/5-HT and DOPAC/DA ratios. More interesting, stocking density had significant effects on brain dopaminergic activity. Fish kept at the lowest density (1 fish/L) displayed higher brain DA concentrations but lower DOPAC/DA ratios than fish kept at 3 fish/L. Fish kept at the lowest density also showed much higher levels of aggression than fish kept at higher density. The DA system is known to be involved in the control of agonistic behavior, and it is usually believed to have a stimulatory effect on aggressive behavior (Winberg and Nilsson, 1992; Summers and Winberg, 2006). However, the fact that fish kept at 1 fish/L showed lower DOPAC/DA rations than fish kept at 3 fish/L makes it difficult to interpret these results. Possibly, higher brain DA levels in fish at the lowest stocking density reflects an upregulation of the DA system. In a similar study, Andersson et al. (2022) analyzed brain monoamines in zebrafish kept at different densities but did not detect any effects of stocking density. However, since metabolites were not assayed it is difficult to draw any conclusions from the results of that study. The fact that we observed effects on the brain DOPAC/DA ratios suggests that stocking density is affecting synaptic release of DA, even though the relationship to agonistic behavior is less clear. We also observed a sex difference in brain 5-HT activity, indicated by lower brain concentrations of both 5-HT and 5-HIAA, as well as lower 5-HIAA/5-HT ratios, in females. Again, it is difficult to speculate on how the difference in brain 5-HT activity between males and females is related to fish behavior and welfare. In zebrafish of the AB strain, males have been reported to be more aggressive than females (Filby et al., 2010; Paull et al., 2010). If so, this may result in more intense social stress in males and, thus, higher brain 5-HT activity. In the previous similar study, brain monoamines were not reported separately for males and females (Andersson et al., 2022).

In this study we did not observe any effects of environmental enrichment, neither of the gravel picture or plastic plants nor of a combination of these enrichments. Still, it has been shown that zebrafish prefer environmental enrichment if given a choice between barren and enriched environments (Schroeder et al., 2014). Moreover, environmental enrichment increases brain size (DePasquale et al., 2016) and proliferation of telencephalic cells in zebrafish (von Krogh et al., 2010). Environmental enrichment also affects zebrafish behavior, decreasing neophobia and anxiety-like behavior (Manuel et al., 2015), as well as decreasing the cortisol response to acute stress in both isolated and group-housed zebrafish (Giacomini et al., 2016). In our study the fish did not seem to use the enrichment as shelter since they made very few attempts of active hiding. For this study we used commercially available plastic plants which seem to be the most common enrichment in zebrafish facilities. However, the design of this enrichment is obviously not optimal. In studies where enrichment have been shown to affect zebrafish brain development, behavior and stress responses the enrichment has been more complex and also included gravel substrate (von Krogh et al., 2010; Manuel et al., 2015; DePasquale et al., 2016; Giacomini et al., 2016). In order to provide shelter, the enrichment needs to create enough three-dimensional structure and, most importantly, not make it possible for the dominant fish to monopolize the enrichment. The use of natural gravel substrate will, for hygienic reasons, not be possible in large zebrafish facilities.

We observed a skewed sex ratio with a high proportion of males. In zebrafish, sex differentiation is believed to be completed at 60 dpf (Takahashi, 1977). Laboratory strains of zebrafish, including the AB strain used in the present study, have lost their master sex-determining gene (Wilson et al., 2014). Instead, these zebrafish strains appear to have a polygenic sex determining system with strong environmental influence (Liew et al., 2012). Keeping zebrafish at high density has been reported to result in a male biased sex ratio (Hazlerigg et al., 2012; Ribas et al., 2017). However, in our experiment the fish were 2 months old at the start of the experiment. Thus, they should have been sexually differentiated already at the start of the experiment. In agreement with this we did not observe any effects of density on the sex ratio.

At the termination of the experiment there were significant effects of fish density on both body mass and total length, fish kept at low density being larger. Thus, growth rate was higher at low densities. Previous studies have also reported that growth is negatively correlated with stocking density (reviewed by Andersson and Kettunen, 2021). However, in most cases the high fish densities, in which growth deprivation was observed, were higher than the stocking densities applied in the present study (Sirn, 2008; Hazlerigg et al., 2012; Rabbane et al., 2017). Moreover, even though Hazlerigg et al. (2012) reported a strong negative correlation between fish density and growth when feed was limited this negative relationship disappeared when feed was provided in proportion to individual requirements. In our study, all groups were fed *ad libitum* and access to feed was not a limiting factor at any of the stocking densities. In addition to lower growth rate at high fish density we also observed higher variability in growth rate, as indicated by elevated CV for body length and body mass. Growth depensation is common in fishes and is assumed to be an effect of agonistic interactions, resulting in uneven food availability with socially dominant fish consuming a larger portion of the group meal (Jobling et al., 1999), but also factors such as group size, feeding ratios, and feeding regimes have been reported to result in higher variance in body mass and body length (Ryer and Olla, 1995; Flood et al., 2012). As shown by the results from behavioral observations of the present study the level of aggression is considerably higher at low stocking densities, at least at densities below 3 fish/L. Thus, the development of strong dominance hierarchies with high levels of overt aggression is unlikely to have caused the high variance in body length and body mass observed at high fish densities in the current study. Zebrafish do not feed from the bottom of the tank (pers. obs.) and in the present study excess feed accumulating on the bottom had to be removed on a regular basis. As a consequence, the feed is only available to the fish during a limited time, when on the surface or sinking. Thus, even though feed was provided in excess the feeding regime, with the feed always provided at the same spot (feeding hole in the lid of the tank), may have resulted in uneven food availability. Fast growing fish may have monopolized access to this feeding spot even if the level of overt aggressive acts was low.

In conclusion, the results of the present study show that keeping zebrafish at densities below 3 fish/L may result in high levels of aggression and the development of strong dominance hierarchies which results in chronic social stress in subordinate fish. However, keeping the fish at high density may also have negative effects on fish performance, as illustrated by lower growth increased growth depensation at high density. Inspection of the fish also becomes more difficult at high density making it difficult to identify sick or injured fish. Based on the results from the current study, the optimal stocking density is likely to be in the range of 3–6 fish/L. However, inter-strain differences in agonistic behavior have been reported and for high aggressive strains higher densities may have to be applied. We did not observe any effects of environmental enrichment. Still, enrichment may have positive welfare effects. However, the design of the environmental enrichment appears crucial.

## Data availability statement

The original contributions presented in this study are included in the article/Supplementary material, further inquiries can be directed to the corresponding author.

## Ethics statement

The animal study was reviewed and approved by the Uppsala Animal Ethical Committee (permit 5.8.18-10125/2018).

## Author contributions

OSS contributed substantially to experimental design, execution, and wrote the first draft of the manuscript. NF did the observation and helped OSS to run water sampling and NTDT. OSS, NF, P-OT, and FA performed analyses of cortisol and brain monoamines. SW and ER obtained funding, supervised the experimental plan, helped with data analysis, and wrote the manuscript. All authors contributed to manuscript revision, read, and approved the submitted version.
