# Supplementary material for: Optimizing zebrafish rearing−Effects of fish density and environmental enrichment
Source: Front Behav Neurosci. 2023 Jun 29;17:1204021. doi: 10.3389/fnbeh.2023.1204021 (PMC10340554; doi:10.3389/fnbeh.2023.1204021)
Supplement: Supplementary file 1 [file Presentation_1.pdf]

Supplementary Material

1 Supplementary Figure

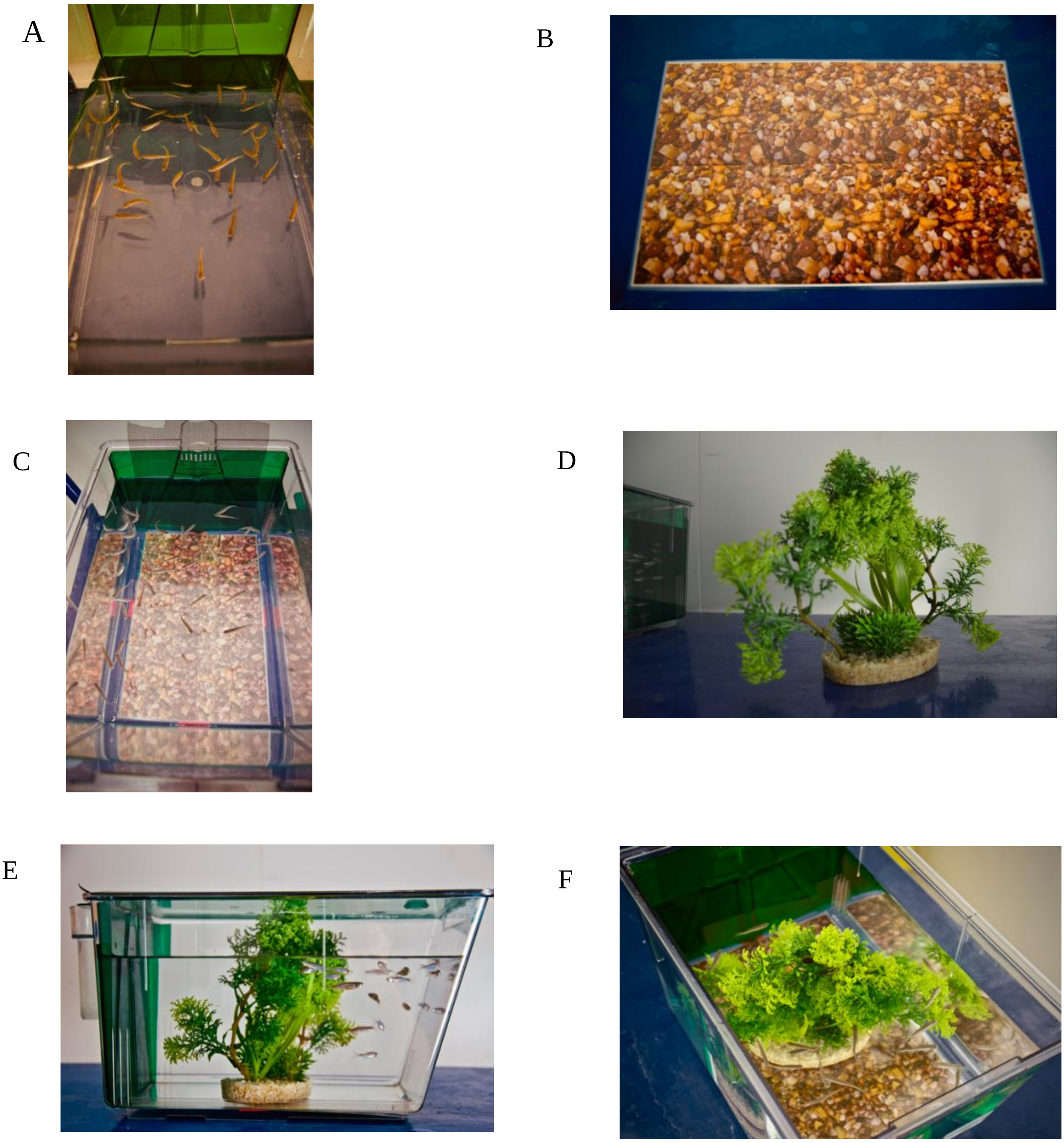

Fig. S1. Pictures showing **(A)** a barren housing tank, **(B)** the gravel picture used as bottom substrate (picture used by Schroeder et al., 2014), **(C)** a housing tank with gravel picture at the bottom, **(D)** an example of a commercially available plastic plant used in this experiment, **(E)** a housing tank with a plant, and **(F)** a housing tank with a combination of plant and gravel picture.

2 Supplementary Tables

Table S1. Ethogram of zebrafish aggression-related behaviours, based on the Zebrafish Neurobehavioral Catalog (ZNC) (Kalueff et al., 2013).

Behaviour	Brief definition
Biting	A fish moves quickly towards another fish and opens and closes its mouth in physical contact with the body surface.
Charging	A fish moves aggressively and fast towards another fish, while the attacked fish avoids the attacker.
Chasing	Active pursuit of the fleeing fish.
Circling	Two fish circle one another repetitively.
Fighting	An aggressive confrontation between two fish, often portrayed as an established dominance.
Fleeing	An attacked fish rapidly swims away from the attacker to escape.
Freezing	A fish stays immobile, often with retracted fins.
Striking	A fish moves aggressively and fast towards another fish with any physical contact.

Table S2. Fulton K (Froese, 2006), body length, body mass and coefficients of variance (CV) of fish kept at different densities and in different environments.

		1 fish/L				3 fish/L				6 fish/L			
		**Tank 1 (n = 35)**	**Tank 2 (n = 37)**	**Tank 3 (n = 39)**	**Tank 4 (n = 37)**	**Tank 1 (n = 115)**	**Tank 2 (n = 115)**	**Tank 3 (n = 104)**	**Tank 4 (n = 108)**	**Tank 1 (n = 213)**	**Tank 2 (n = 219)**	**Tank 3 (n = 208)**	**Tank 4 (n = 212)**
Barren	Fulton K	1.7±0.2	1.8±0.2	2.1±0.3	1.9±0.3	1.7±0.2	1.8±0.3	1.7±0.3	1.8±0.3	1.6±0.3	1.7±0.2	1.6±0.4	1.7±0.3
	CV of Fulton K (%)	8.8	11.5	13.8	16.3	10.9	18.4	16.9	15.5	19.0	15.0	26.6	16.9
	Body length (cm)	2.7±0.2	2.9±0.2	2.7±0.1	2.9±0.4	2.4±0.2	2.4±0.3	2.5±0.2	2.4±0.2	2.2±0.3	2.2±0.3	2.3±0.3	2.4±0.4
	CV of body length (%)	7.8	7.4	3.5	12.5	10.5	10.4	6.8	8.4	14.0	12.3	13.8	15.3
	Body mass (g)	0.32±0.07	0.43±0.09	0.4±0.05	0.48±0.2	0.23±0.07	0.26±0.09	0.26±0.05	0.25±0.07	0.19±0.08	0.19±0.07	0.20±0.08	0.23±0.08
	CV of body mass (%)	22.9	20.1	12.9	41.3	30.9	35.8	17.6	26.3	40.6	35.1	40.6	35.9
Gravel picture	Fulton K	1.7±0	1.8±0	2±0	1.9±0	1.6±0	1.7±0	2±0	1.8±0	1.7±0	1.7±0	1.7±0	1.7±0
	CV of Fulton K (%)	13.7	13.5	10.5	20.5	20.3	11.7	15.5	19.6	17.0	18.9	17.2	14.9
	Body length (cm)	2.7±0.1	2.7±0.2	2.6±0.1	2.8±0.3	2.4±0.2	2.4±0.2	2.4±0.3	2.5±0.2	2.3±0.3	2.3±0.3	2.3±0.3	2.2±0.2
	CV of body length (%)	5.1	8.3	5.3	9.1	8.8	9.4	11.0	8.9	12.5	12.6	11.2	9.9
	Body mass (g)	0.35±0.05	0.37±0.07	0.35±0.04	0.44±0.18	0.23±0.07	0.24±0.06	0.28±0.08	0.27±0.08	0.22±0.08	0.20±0.06	0.20±0.06	0.20±0.06
	CV of body mass (%)	15.7	19.8	13.0	41.3	29.1	25.1	28.4	31.2	35.6	30.9	30.5	30.3
Gravel picture + plastic plant	Fulton K	1.8±0.3	2±0.4	1.8±0.2	1.6±0.5	1.8±0.3	1.7±0.2	1.6±0.3	1.7±0.2	1.6±0.2	1.7±0.3	1.6±0.2	2±3
	CV of Fulton K (%)	18.6	20.2	10.5	28.1	14.8	12.5	17.4	12.2	13.0	16.0	14.6	146.6
	Body length (cm)	2.8±0.2	3±0.3	2.6±0.3	2.9±0.3	2.3±0.2	2.4±0.2	2.4±0.2	2.4±0.2	2.3±0.2	2.3±0.3	2.3±0.2	2.3±0.2
	CV of body length (%)	8.1	9.8	10.5	11.7	9.1	7.2	9.0	8.3	9.5	12.5	9.1	10.0
	Body mass (g)	0.4±0.13	0.51±0.16	0.33±0.1	0.36±0.07	0.23±0.06	0.25±0.05	0.24±0.07	0.26±0.07	0.20±0.05	0.21±0.06	0.20±0.06	0.23±0.24
	CV of body mass (%)	32.0	32.2	29.4	20.5	24.4	19.2	29.5	27.8	26.9	28.7	30.0	103.2
Plastic plant	Fulton K	1.8±0.2	1.9±0.2	1.8±0.3	2±0.4	1.7±0.2	1.9±0.3	1.7±0.2	1.7±0.2	1.7±0.2	1.7±0.2	1.7±0.2	1.7±0.4
	CV of Fulton K (%)	10.7	8.2	17.6	19.9	13.3	15.4	13.5	11.0	13.1	13.8	12.8	22.4
	Body length (cm)	2.6±0.2	2.8±0.3	2.9±0.3	2.8±0.2	2.5±0.2	2.4±0.2	2.4±0.3	2.4±0.2	2.3±0.2	2.3±0.2	2.3±0.3	2.3±0.3
	CV of body length (%)	8.4	9.3	11.0	7.7	8.9	6.9	11.3	7.4	9.2	9.6	11.9	11.6
	Body mass (g)	0.34±0.08	0.42±0.1	0.43±0.08	0.42±0.03	0.26±0.07	0.25±0.06	0.24±0.07	0.24±0.06	0.22±0.06	0.21±0.06	0.21±0.07	0.22±0.09
	CV of body mass (%)	24.1	24.0	18.2	8.2	26.9	25.7	27.4	23.4	27.0	29.3	31.9	40.2

Data are presented as mean±SD.

Table S3. Results from the novel tank diving test in zebrafish kept at different densities (1 fish/L, 3 fish/L or 6 fish/L).

		**1 fish/L (n = 61)**	**3 fish/L (n = 59)**	**6 fish/L (n = 61)**
Bottom	Velocity	25.6 (11.1)	23.0 (10.6)	22.6 (12.4)
	Distance moved	9036.8 (6451.2)	8838.6 (6399.9)	7195.4 (5172.9)
	Frequency	28.0 (29.0)*	22.0 (23.5)	20.0 (23.0)
	Duration	405.0 (244.4)	444.1 (252.1)	418.2 (275.9)
	%Duration	67.5 (40.7)	74.0 (42.0)	69.7 (46.0)
Middle	Velocity	33.8 (10.9)	36.7 (16.0)	33.6 (15.3)
	Distance moved	4450.6 (4685.2)	3504.2 (4129.3)	2522.2 (4298.6)
	Frequency	51.0 (49.0)*	37.0 (40.5)	29.0 (43.0)
	Duration	120.2 (129.6)	105.7 (148.7)	105.5 (133.9)
	%Duration	20.0 (21.6)	17.6 (24.8)	17.6 (22.3)
Top	Velocity	37.3 (14.9)	35.3 (22.6)	35.2 (15.8)
	Distance moved	2548.8 (4646.1)	2023.7 (3059.8)	2722.2 (4909.5)
	Frequency	16.0 (27.0)	10.0 (21.0)	8.0 (22.0)
	Duration	65.0 (121.6)	36.5 (94.4)	58.4 (141.0)
	%Duration	10.8 (20.3)	6.1 (15.7)	9.7 (23.5)
Arena	Velocity	28.0 (10.7)	24.6 (10.1)	25.5 (13.9)
	Distance moved	16630.7 (6401.5)	14741.5 (6058.0)	15293.9 (8325.6)
	Total activity	101.0 (98.0)*	75.0 (81.5)	59.0 (88.0)

Values are presented as median with interquartile range in brackets. *$p < 0.05$ compared to fish kept at 3 or 6 fish/L (Mann-Whitney U-test).

Table S4. Results from the novel tank diving test in zebrafish kept in barren tanks or in tanks with different enrichments.

		**Barren (n = 47)**	**Gravel picture (n = 47)**	**Gravel picture + plant (n = 45)**	**Plant (n = 42)**
Bottom	Velocity	23.1 (12.8)	24.3 (11.7)	24.6 (9.7)	21.7 (12.9)
	Distance moved	6780.0 (6323.7)	8772.2 (6385.6)	9784.1 (6552.2)	8537.3 (5727.7)
	Frequency	27.0 (24.5)	27.0 (28.0)	24.0 (18.0)	19.5 (25.0)
	Duration	405.0 (302.4)	351.2 (328.8)	418.2 (234.4)	463.1 (204.5)
	%Duration	67.5 (50.4)	58.5 (54.8)	69.7 (39.1)	77.2 (34.1)
Middle	Velocity	33.4 (13.8)	36.9 (18.1)	32.8 (13.1)	32.5 (13.2)
	Distance moved	3919.5 (4646.2)	3664.4 (4903.7)	3251.7 (3700.3)	3077.2 (4100.2)
	Frequency	42.0 (51.5)	52.0 46.0)	37.0 (37.0)	30.5 (43.5)
	Duration	122.5 (143.4)	113.9 (165.4)	105.7 (125.0)	80.3 (118.4)
	%Duration	20.4 (23.9)	19.0 (27.6)	17.6 (20.8)	13.4 (19.7)
Top	Velocity	31.2 (25.8)	38.1 (26.5)	29.3 (14.8)	33.4 (19.0)
	Distance moved	2038.8 (4807.9)	2008.6 (5735.0)	1648.5 (3454.6)	1537.0 (3257.7)
	Frequency	13.0 (30.5)	18.0 (25.5)	12.0 (20.0)	8.5 (19.5)
	Duration	57.1 (135.8)	62.5 (148.1)	58.6 (114.3)	42.8 (84.1)
	%Duration	9.5 (22.6)	10.4 (24.7)	9.8 (19.0)	7.1 (14.0)
Arena	Velocity	26.6 (13.4)	26.7 (13.1)	26.1 (10.5)	25.6 (14.4)
	Distance moved	15956.2 (8067.5)	16038.7 (7829.9)	15661.3 (6293.4)	15345.7 (8653.4)
	Total activity	85.0 (103.0)	105.0 (92.5)	75.0 (74.0)	60.5 (85.8)

Values are presented as median with interquartile range in brackets. There were no significant differences of the enrichment.

Supplementary Table 5. Results from the novel tank diving test in zebrafish exposed to individual confinement stress in a 50 mL Falcon tube for 30 min or no stress 18–27 days prior to the behavioural testing.

		**No stress (n = 96)**	**Stress (n = 84)**
Bottom	Velocity	22.9 (10.2)	24.3 (13.9)
	Distance moved	9078.7 (5659.5)	8018.9 (6527.9)
	Frequency	21.0 (25.8)	26.0 (24.0)
	Duration	471.5 (256.6)	377.4 (246.5)*
	%Duration	78.6 (42.8)	62.9 (41.1)*
Middle	Velocity	32.4 (14.5)	36.1 (14.0)
	Distance moved	2294.0 (4729.3)	4338.5 (3914.2)*
	Frequency	29.0 (46.5)	48.0 (42.3)*
	Duration	77.9 (141.9)	128.5 (124.9)*
	%Duration	13.0 (23.6)	21.4 (20.8)*
Top	Velocity	29.6 (21.9)	35.7 (16.7)*
	Distance moved	1012.7 (3614.8)	2414.9 (4307.7)*
	Frequency	8.0 (20.3)	18.0 (22.5)*
	Duration	36.0 (109.5)	65.5 (124.9)*
	%Duration	6.0 (18.2)	10.9 (20.8)*
Arena	Velocity	25.2 (10.4)	27.2 (14.9)
	Distance moved	15106.9 (6264.7)	16299.0 (8918.5)
	Total activity	58.5 (92.8)	95.5 (85.3)*

Values are presented as median with interquartile range in brackets. * $p < 0.05$ compared to non-stressed fish (Mann-Whitney U-test).

3 Supplementary Information

Liquid Chromatography-Mass Spectrometry (LC-MS)
Samples were mixed with 20% isopropanol due to the cortisol 1.6 LogP3 value.
Sample Manager FTN-H parameters: injection volume: 10 l, auto-sampler temperature: 25°C, column temperature: 50°C, Column ACQUITY UPLC® BEH C18, 1.7 m, 2.1 x 50 mm, needle wash: 50% H2O and 50% acetonitrile.
Quaternary Solvent Manager parameters: seal wash 90% H2O and 10% acetonitrile, purge wash 90% H2O and 10% acetonitrile, pump flow 0.5 mL/min.
Mobile phase A: 90% H2O, 10% acetonitrile, and 0.1% formic acid.
Mobile phase B: 90% acetonitrile, 10% H20, and 0.1% formic acid.
A linear gradient elution between the mobile phases began at an initial concentration of solvents A:B of 90:10, ending with A:B of 0:100, before shifting back to the initial concentration.
Mobile phase gradient:
1 minute: gradient ratio A:B 90:10
3.5 minutes: gradient ratio A:B 0:100
4 minutes: gradient ratio A:B 0:100
End of run at 4.2 minutes: gradient ratio A:B 90:10
End of post time run at 6.1 minutes: gradient ratio A:B 90:10
QDa detector parameters: SIR mode 407.25 Da with negative cone voltage of 15 V. Sampling rate of 8 points/sec and capillary voltage of 800 V.

4 References

Froese, R. (2006). Cube law, condition factor and weight–length relationships: history, meta-analysis and recommendations. J Appl Ichthyol 22:241–253. doi: 10.1111/j.1439-0426.2006.00805.x.

Kalueff, A. V., Gebhardt, M., Stewart, A. M., Cachat, J. M., Brimmer, M., Chawla, J. S., et al. (2013). Towards a Comprehensive Catalog of Zebrafish Behavior 1.0 and beyond. Zebrafish 10, 70–86. doi: 10.1089/zeb.2012.0861.

Schroeder, P., Jones, S., Young, I. S., and Sneddon, L. U. (2014). What do zebrafish want? Impact of social grouping, dominance and gender on preference for enrichment. Lab Anim 48, 328–337. doi: 10.1177/0023677214538239.
